# Sex-dependent alterations of salivary microbiome in Parkinson’s disease: associations with motor and non-motor clinical phenotypes

**DOI:** 10.3389/fmolb.2025.1726620

**Published:** 2025-12-08

**Authors:** Jie Zu, Wei Zhang, Li Du, Hui Zhao, Min Xu, Ruyi Chen, Yuting Zhang, Siyan Chen, Chuanying Xu, Liguo Dong, Jienan Zhu, Lishun Xiao, Chunfeng Liu

**Affiliations:** 1 Department of Neurology, The Second Affiliated Hospital of Soochow University, Suzhou, China; 2 Department of Neurology, The Affiliated Hospital of Xuzhou Medical University, Xuzhou, China; 3 Department of Biostatistics, School of Public Health, Xuzhou Medical University, Xuzhou, China

**Keywords:** Parkinson’s disease, saliva, oral microbiome, sex differences, 16S rRNA sequencing, UPDRS

## Abstract

**Background:**

Parkinson’s disease (PD) shows considerable heterogeneity in motor and non motor features. The contribution of the salivary microbiome and its modification by sex remains unclear.

**Methods:**

In a single center cross sectional case control study, we profiled unstimulated saliva from 24 patients with Parkinson’s disease and 25 age and sex matched controls using 16S rRNA sequencing. Alpha and beta diversity were evaluated, group associated taxa were identified by indicator analysis, and community structure was related to clinical measures including Unified Parkinson’s Disease Rating Scale part III in off and on medication states, the Non Motor Symptoms Scale, and the Hamilton Depression Rating Scale.

**Results:**

Alpha diversity was broadly preserved, whereas richness was higher in men with Parkinson’s disease than in women with PD. Beta diversity showed modest but significant separation across disease by sex groups at multiple taxonomic levels with PERMANOVA *R* ^2^ about 0.13 and significant *P* values. Women with PD displayed higher *Prevotella* and *Veillonella* with lower *Akkermansia*, and men with PD showed a TM7 skewed profile typified by *Candidatus Saccharimonas* and reduced *Haemophilus*. The coupling between community structure and clinical burden was strongest for motor severity and was more evident in the on medication state.

**Conclusion:**

The salivary microbiome in Parkinson’s disease exhibits sex specific alterations that track clinical burden, supporting sex aware development of salivary biomarkers and microbiota focused strategies. Validation in larger longitudinal cohorts with multi omics and standardized oral and medication metadata is warranted.

## 1 Introduction

Parkinson’s disease (PD) is a common neurodegenerative disorder. It causes motor impairment and many non-motor problems (Tanner and Ostrem, 2024). Beyond classical nigrostriatal degeneration involving the loss of dopaminergic neurons between the substantia nigra and the striatum, and α-synuclein pathology, host microbiome interactions may contribute to PD through immune, metabolic, and neural pathways (Wallen et al., 2022; Claudino Dos Santos et al., 2023). In addition to the established links between microbial dysbiosis and central nervous system disorders, recent work has expanded the concept of health associated oral and gut microbiota species that may contribute to systemic homeostasis (Frey et al., 2018). Altered gut communities are well described, whereas the salivary microbiome remains comparatively underexplored in this context (Berthouzoz et al., 2023; Stagaman et al., 2024).

Oral problems are common in PD, including dysphagia, sialorrhea, periodontal disease, and altered salivary flow, all of which can collectively reshape the salivary microbial ecology (Verhoeff et al., 2023; Qiu et al., 2025; Isaacson et al., 2020). Early reports suggest that salivary communities differ between patients and healthy controls (Stagaman et al., 2024; Yay et al., 2023). Patterns point to enrichment of anaerobic genera such as Prevotella, *Fusobacterium*, and Veillonella, alongside depletion of barrier-supporting commensals including *Haemophilus* and *Neisseria* (Yay et al., 2023; Fleury et al., 2021; Clasen et al., 2025). Such shifts may promote mucosal inflammation, acidogenic metabolism and epithelial stress (Rozas et al., 2021). These processes could link the oral niche to systemic and neural changes that matter for PD. Most studies did not test whether sex modifies these links.

Sex is a major source of heterogeneity in PD (Zirra et al., 2023). Men have higher incidence and often heavier motor burden (Maas et al., 2024). Women often report more non-motor and affective symptoms (Cattaneo and Pagonabarraga, 2025). Sex hormones, immune tone and oral mucosal biology differ between men and women (Del Pinto et al., 2024). These factors can shape microbial composition and host responses (Hamilton et al., 2024). It is therefore plausible that salivary microbiome–phenotype relationships are sex specific (Hokanson et al., 2024). Few studies have examined this question with direct links to motor and non-motor scales.

The aim of this study was to address this gap. We profiled saliva from patients with PD and matched healthy controls using 16S rRNA sequencing. We compared diversity and composition and identified group associated taxa. We then related microbial patterns to clinical measures. Motor severity was assessed using the Unified Parkinson’s Disease Rating Scale (UPDRS) (Goetz et al., 2008), non-motor burden using the Non-Motor Symptoms Scale (NMSS) (Chaudhuri et al., 2007), and affective symptoms using the Hamilton Depression Rating Scale (HAMD) (Hamilton, 1960). We hypothesised that patients show altered salivary communities and that the associations between microbiota and clinical features differ by sex. Our goal was to clarify the role of the oral microbiome in disease heterogeneity and to support development of sex aware salivary biomarkers and interventions.

## 2 Materials and methods

### 2.1 Overview of the study workflow

**FIGURE 1:**
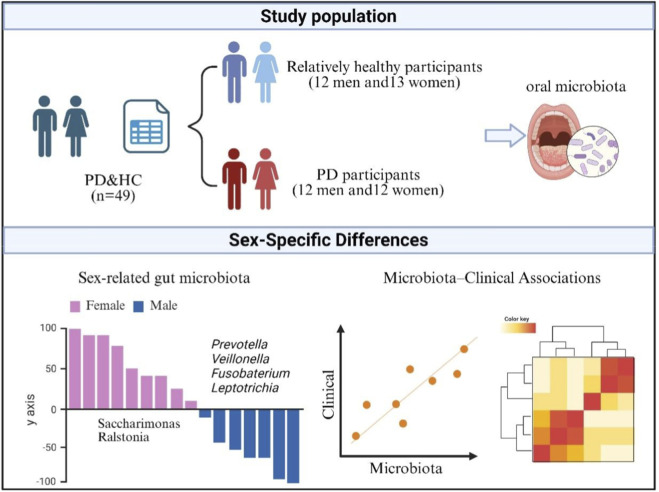
Summary of the study population, sex-specific salivary microbiome differences, and microbiota–clinical associations. The study included 49 participants comprising PD patients (12 men and 12 women) and healthy controls (HC; 12 men and 13 women). Unstimulated saliva samples were collected for oral microbiota profiling. Sex-stratified analyses revealed distinct microbial signatures, with *Prevotella*, *Veillonella*, *Fusobacterium*, and *Leptotrichia* enriched in women, and *Saccharimonas* and *Ralstonia* enriched in men. Associations between salivary microbiome composition and clinical measures were examined, demonstrating links between sex-dependent microbial variation and symptom severity.

The study followed an integrated workflow that encompassed participant recruitment, standardized collection of unstimulated saliva, microbial DNA extraction, profiling of bacterial communities through 16S rRNA gene sequencing of the V4–V5 region, and bioinformatic processing to generate high-quality amplicon sequence variants. These data were subsequently used to perform sex-stratified taxonomic analyses and to examine associations between salivary microbiome composition and clinical measures, including motor and non-motor symptom severity (Figure 1).

### 2.2 Study population

We conducted a cross sectional case control study. We recruited forty nine participants from the Department of Neurology at Xuzhou Medical University Affiliated Hospital. The PD group included twenty-four patients (12 women and 12 men). The healthy control (HC) group included twenty-five participants (13 women and 12 men) who were individually matched by age and sex during enrollment. One additional female volunteer who fulfilled all inclusion criteria and passed quality control was retained, as her data were valid and within the approved sample size. Demographic and baseline clinical characteristics of the study population, stratified by sex and disease status, are summarized in Table 1. The diagnosis of PD followed the Movement Disorder Society clinical diagnostic criteria. Inclusion criteria were age from forty five to 75 years and Hoehn and Yahr stage from one to three. Exclusion criteria were use of antibiotics or probiotics within 1 month, active oral or systemic infection, major neurological or psychiatric comorbidity, and use of immunosuppressive drugs. The Ethics Committee of Xuzhou Medical University Affiliated Hospital approved the protocol(XYFY2023-KL473-01). All participants gave written informed consent.

**TABLE 1:** Demographic and clinical characteristics.

Variable	PD_Male (n = 12)	PD_Female (n = 12)	HC_Male (n = 12)	HC_Female (n = 13)	p
Age (years)	68.3 ± 5.4	67.9 ± 6.0	68.0 ± 5.0	67.7 ± 5.5	0.72
UPDRS part I score	13.75 ± 0.89	12.33 ± 0.99	–	–	0.30
UPDRS part II score	22.50 ± 4.40	16.92 ± 3.50	–	–	0.33
UPDRS part III score(off-state)	71.58 ± 7.71	40.75 ± 5.18	–	–	0.0031
UPDRS part III score(on-state)	63.00 ± 7.00	31.25 ± 3.01			0.0004
HAMD	13.92 ± 1.89	31.50 ± 4.38			0.0013
NMSS	25.33 ± 4.93	43.75 ± 6.61			0.0361

### 2.3 Clinical assessments

Motor severity was evaluated using the UPDRS part III in both off- and on-medication states, which quantifies motor symptoms such as tremor, rigidity, and bradykinesia (Goetz et al., 2008). Non-motor symptoms were assessed using the NMSS, which evaluates 30 items across domains including sleep, autonomic function, cognition, and mood (Chaudhuri et al., 2007). Depressive symptoms were measured using the HAMD, a 17-item clinician-rated instrument that assesses the severity of depression across mood, guilt, anxiety, and somatic features (Hamilton, 1960). Demographic and clinical information was obtained through structured interviews and review of medical records. Oral health status was screened at enrollment by interview and brief intra-oral inspection. Participants with current acute oral infections, recent tooth extraction or major dental procedures (within the past 3 months), ongoing systemic antibiotic therapy, or self-reported severe periodontal disease were not included. Obvious oral ulcers, abscesses, or purulent gingival lesions on inspection were considered exclusion criteria.

### 2.4 Saliva collection and DNA extraction

Unstimulated whole saliva (∼5 mL) was collected from each participant between 8:00 and 10:00 a.m. after at least 2 hours of fasting from food or beverages other than water, smoking, or oral hygiene activities. Samples were immediately placed on ice, transported to the laboratory within 2 hours, aliquoted, and stored at −80 °C until further processing. Microbial DNA was extracted using the QIAamp DNA Microbiome Kit (Qiagen, Germany) according to the manufacturer’s protocol. DNA concentration and purity were assessed with a NanoDrop spectrophotometer (Thermo Fisher Scientific, United States).

### 2.5 S rRNA gene sequencing and bioinformparagraphatic analysis

Bacterial community composition was profiled by amplifying the V4–V5 region of the 16S rRNA gene using primers 515F (5′-GTGCCAGCMGCCGCGG-3′) and 907R (5′-CCGTCAATTCMTTTRAGTTT-3′), following established protocols for bacterial community profiling (Caporaso et al., 2011). Amplicon libraries were purified and sequenced on the Ion S5 platform (Thermo Fisher Scientific, United States). Raw sequencing data were demultiplexed and trimmed with Cutadapt, quality-filtered (Q ≥ 30), and denoised using the DADA2 algorithm implemented in QIIME2 (v2023.2). Chimeric sequences were removed, and high-quality amplicon sequence variants (ASVs) were assigned taxonomy against the SILVA v138 reference database using a confidence threshold of 0.8. Downstream analyses included estimation of α-diversity (Sobs, ACE, Shannon, and Simpson indices) and β-diversity (Bray-Curtis dissimilarities) with QIIME2 and R (v4.3.1). Community-level differences were evaluated by PERMANOVA, and differentially abundant taxa were identified using Kruskal–Wallis tests with false discovery rate (FDR) correction. This workflow follows established guidelines for microbiome profiling and reporting (Sackett et al., 2022).

### 2.6 Indicator species analysis

We used the R package labdsv to perform Indicator Value analysis. We calculated indicator values for each taxon from its relative abundance and frequency within groups. We tested significance with 999 permutation iterations. We included only taxa with relative abundance above 0.1 percent at the tested level. We used ten by ten fold cross validation to evaluate robustness. We summarized results at the phylum and genus levels with bubble plots.

### 2.7 Indicator species analysis

Indicator species (IndVal) analysis was used to identify taxa that were both specific to and consistently present within each sex-by-disease group. This approach was selected because it evaluates both specificity and fidelity, which makes it well suited for detecting group-characterizing taxa in ecological studies. Other differential abundance tools, such as DESeq2, LEfSe, or ANCOM, focus primarily on mean abundance differences and are less effective for identifying taxa that define ecological group identity. Statistical significance was assessed using permutation tests, and all p-values were adjusted using the Benjamini–Hochberg false discovery rate.

### 2.8 Mantel test

Mantel analyses were performed using the vegan package in R. Bray–Curtis dissimilarity matrices of the salivary microbiome were compared with Euclidean distance matrices derived from clinical variables, including UPDRS part III (off and on medication), the NMSS, and the HAMD. All clinical variables were z-standardized before distance computation to ensure comparability across scales. Mantel correlations were estimated using 9,999 permutations. Correlation strength was interpreted according to established ecological conventions, with r values below 0.10 considered very weak, 0.10 to 0.30 weak, 0.30 to 0.50 moderate, and values above 0.50 strong. Results were visualized using correlation diagrams and heatmaps and stratified by sex.

### 2.9 Statistical analysis

**FIGURE 2:**
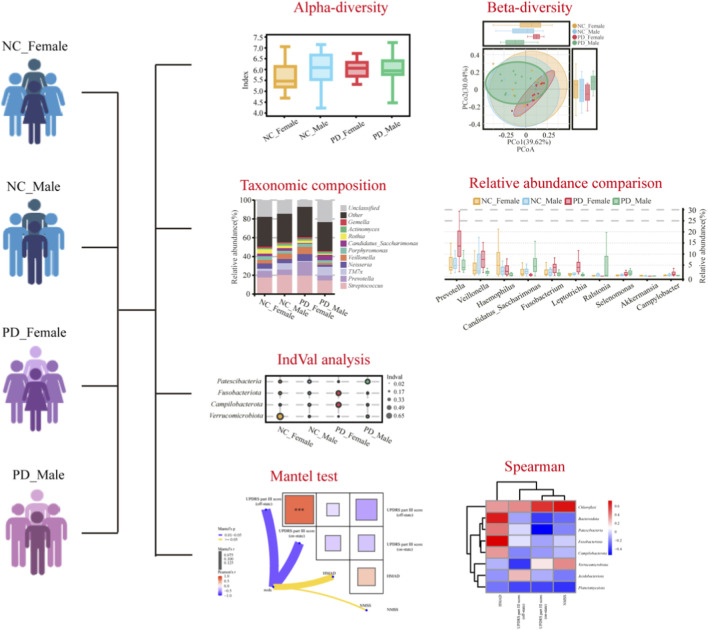
Overview of study design and analytical workflow. The schematic summarizes the sequential design of this study. Unstimulated saliva samples were collected from 24 patients with PD and 25 age- and sex-matched HC. Microbial DNA was extracted and subjected to 16S rRNA V4–V5 region amplification using primers 515F and 907R. Amplicons were sequenced on the Ion S5 platform, and bioinformatic processing was performed using the QIIME2–DADA2 pipeline for denoising, chimera removal, and taxonomy assignment against the SILVA v138 database. Alpha and beta diversity metrics were computed, and differential taxa were identified using Kruskal–Wallis tests with FDR correction. Indicator value (IndVal) and Mantel analyses were used to relate microbial community structure to clinical indices including UPDRS, NMSS, and HAMD.

We performed statistical analysis in R version 4.3.1. We compared continuous variables using the Student’s t-test or the Mann Whitney U test as appropriate. We compared alpha diversity indices using the Kruskal Wallis test with Dunn post hoc correction. We tested beta diversity differences using PERMANOVA with the vegan package. PERMANOVA estimates the proportion of variance in community composition that is explained by grouping factors. We used permutation with 9,999 iterations. We considered p values less than 0.05 as significant. We combined principal coordinates analysis with PERMANOVA to visualize and test between group differences in overall salivary community structure. We identified differentially abundant taxa using the Kruskal Wallis test with false discovery rate correction. We used Spearman correlation to assess associations between microbial abundances and clinical scores. We defined significance as false discovery rate adjusted p less than 0.05. The overall workflow of experimental design and statistical analyses is summarized in Figure 2.

## 3 Results

### 3.1 Demographic and clinical characteristics

**FIGURE 3:**
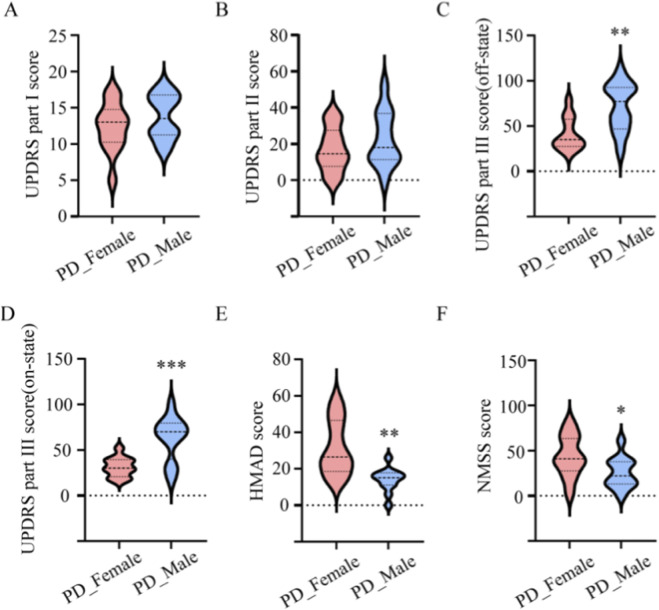
Sex-specific differences in clinical scores among PD patients. Violin plots compare PD_Female and PD_Male groups (n = 12 per sex) for **(A)** UPDRS part I, **(B)** UPDRS part II, **(C)** UPDRS part III OFF medication, **(D)** UPDRS part III ON medication, **(E)** HAMD, and **(F)** NMSS. Data are shown as violin distributions; horizontal dashed lines denote medians; unpaired two-tailed t-tests. *p < 0.05 **p < 0.01, ***p < 0.001.

We enrolled 49 participants. The PD group included 24 patients, 12 women and 12 men. The HC group included 25 individuals, 13 women and 12 men. Groups were comparable in age with a mean close to 68 years. No significant differences were observed in baseline demographics (Table 1). Within the PD cohort, sex stratified analyses showed no difference between men and women for UPDRS part I (13.75 ± 0.89 vs. 12.33 ± 0.99; p = 0.30) and UPDRS part II (22.50 ± 4.40 vs. 16.92 ± 3.50; p = 0.33) (Figures 3A,B). Men had higher UPDRS part III scores in the off medication state (71.58 ± 7.71 vs. 40.75 ± 5.18; p = 0.0031) and in the on medication state (63.00 ± 7.00 vs. 31.25 ± 3.01; p = 0.0004) (Figures 3C,D). Women had higher NMSS (43.75 ± 6.61 vs. 25.33 ± 4.93; p = 0.0361) and HAMD (31.50 ± 4.38 vs. 13.92 ± 1.89; p = 0.0013) (Figures 3E,F). These contrasts defined the clinical context for subsequent microbiome analyses.

### 3.2 Alterations in alpha diversity of salivary microbiota

**FIGURE 4:**
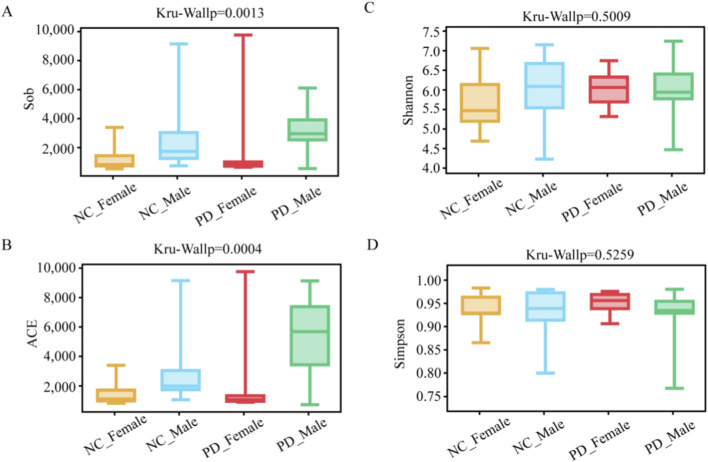
Alpha-diversity of the salivary microbiome by disease status and sex. Panels show **(A)** observed species richness (Sobs), **(B)** ACE richness estimator, **(C)** Shannon diversity, and **(D)** Simpson index across four groups: PD_Male (n = 12), PD_Female (n = 12), HC_Male (n = 12), and HC_Female (n = 13). Data are presented as box-and-whisker plots (center line = median; box = interquartile range; whiskers = range; points = individual samples). Group comparisons were performed using Kruskal–Wallis tests followed by Dunn’s *post hoc* correction for multiple comparisons. Asterisks denote significance levels (*p < 0.05; **p < 0.01; **p < 0.001).

We assessed richness with Sobs and ACE and diversity with Shannon and Simpson by Kruskal–Wallis with Dunn’s post hoc tests (Supplementary Table S1). Global comparison across the four groups showed significant differences in richness (Sobs: p = 0.0013; ACE: p = 0.0004). Pairwise tests indicated higher richness in PD_Male group than in PD_Female group (Sobs: p = 0.0058; ACE: 0.0025) and than in NC_Female group (Sobs: p = 0.0132; ACE: 0.0033). PD_Male group and NC comparisons did not differ. Within each sex, comparisons between PD and control were not significant. Shannon and Simpson indices were broadly similar across groups (Shannon: p = 0.5009; Simpson: p = 0.5259) (Figures 4A–D). These data suggest that salivary alpha diversity *per se* is preserved in PD, whereas species richness shows a sex-related pattern (male > female), with PD_Male exhibiting the highest richness.

### 3.3 Sex-dependent differences in beta diversity of the salivary microbiome

**FIGURE 5:**
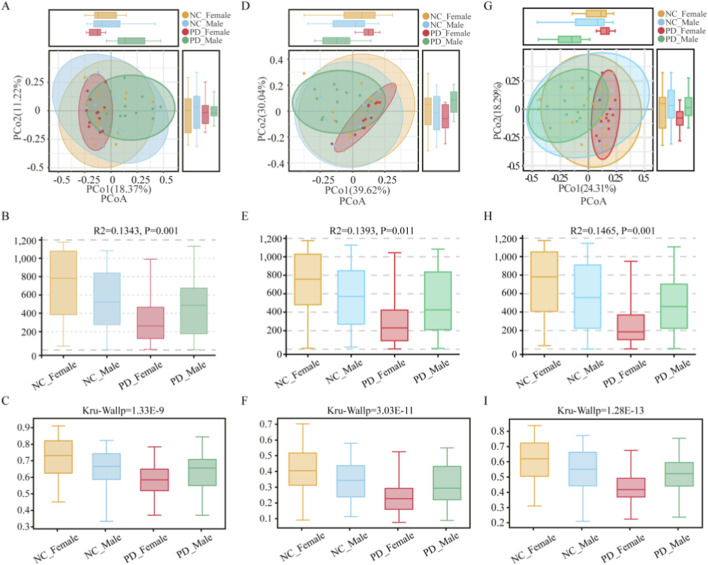
Beta-diversity of the salivary microbiome across PD and HC groups stratified by sex. Panels summarize between-group differences in community composition based on Bray–Curtis distances at three taxonomic levels. **(A–C)** OTU level: **(A)** Principal Coordinates Analysis (PCoA) plot showing partial clustering of samples by disease-by-sex group; **(B)** summary of PERMANOVA (Adonis) results indicating the proportion of variance (*R* ^2^ = 0.134, p = 0.001) explained by grouping; **(C)** boxplots displaying per-sample distances to group centroids. **(D–F)** Phylum level: **(D)** PCoA plot; **(E)** PERMANOVA summary (*R* ^2^ = 0.139, p = 0.011); **(F)** boxplots of within-group dispersion. **(G–I)** Genus level: **(G)** PCoA plot; **(H)** PERMANOVA summary (*R* ^2^ = 0.146, p = 0.001); **(I)** boxplots showing distances to centroids.

Beta diversity analyses using Bray–Curtis distances revealed modest yet statistically significant differences in salivary microbiota composition among sex-stratified PD and HC groups. At the OTU level (Figures 5A–C), PCoA showed partially overlapping clusters and Adonis/PERMANOVA indicated a between-group effect (*R* ^2^ = 0.134, p = 0.001), with concordant Kruskal–Wallis statistics (p = 1.33 × 10^−9^). At the phylum level (Figures 5D–F), differences persisted (*R* ^2^ = 0.139, p = 0.011; Kruskal–Wallis p = 3.03 × 10^−11^). At the genus level (Figures 5G–I), compositional shifts were also evident (*R* ^2^ = 0.146, p = 0.001; Kruskal–Wallis p = 1.28 × 10^−13^). Collectively, these OTU-, phylum-, and genus-level results indicate consistent, statistically significant variation in salivary community structure across the disease-by-sex groups.

### 3.4 Sex-specific taxonomic alterations in salivary microbiota

**FIGURE 6:**
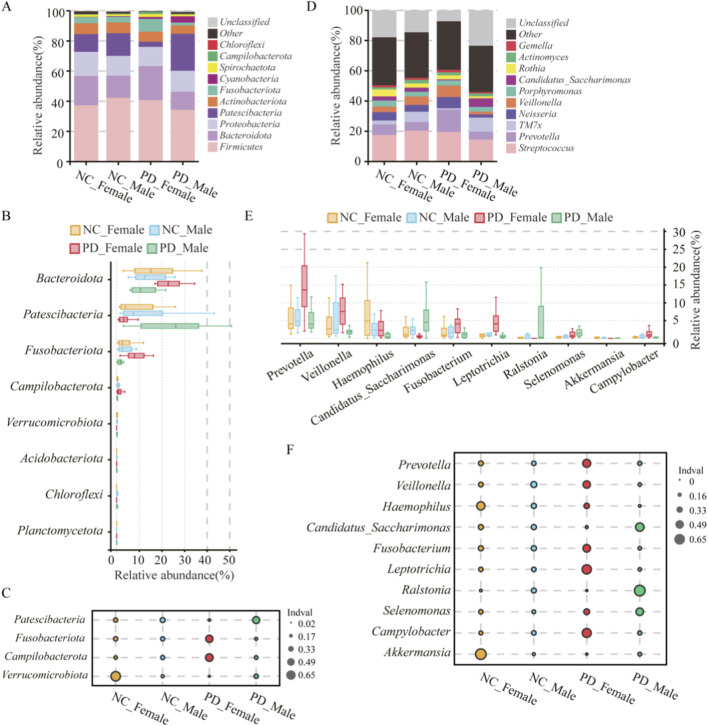
Sex-specific taxonomic composition and indicator taxa of the salivary microbiome in PD and HC. **(A–C)** Phylum level: **(A)** Stacked barplots show mean relative abundance of dominant bacterial phyla across PD_Male (n = 12), PD_Female (n = 12), HC_Male (n = 12), and HC_Female (n = 13) groups. **(B)** Boxplots display per-sample relative abundances for significantly different phyla; **(C)** indicator value (IndVal) analysis identifies phyla most strongly associated with each group. **(D–F)** Genus level: **(D)** Stacked barplots depict mean relative abundances of dominant genera; **(E)** boxplots summarize relative abundances of selected genera differing across groups; **(F)** IndVal analysis identifies group-associated genera. Statistical comparisons were conducted using Kruskal–Wallis tests followed by Dunn’s *post hoc* correction for multiple testing. Asterisks indicate significance levels (*p < 0.05; **p < 0.01; **p < 0.001).

In sex stratified analyses, the PD_Female group and the PD_Male group showed clear taxonomic shifts. At the phylum level, the PD_Female group had higher *Bacteroidota* and showed enrichment of *Fusobacteriota* and *Campylobacterota*. The PD_Male group showed enrichment of *Patescibacteria* and higher *Chloroflexi* and *Planctomycetota*. *Verrucomicrobiota* had the lowest relative abundance in the PD_Female group (Figures 6A,B; Supplementary Table S2). At the genus level, the PD_Female group had higher *Prevotella*, *Veillonella*, *Fusobacterium*, *Leptotrichia*, and *Campylobacter* and lower *Akkermansia*. The PD_Male group had higher *Candidatus_Saccharimonas* and *Ralstonia* and lower *Haemophilus* (Figures 6D,E; Supplementary Table S3). Indicator value analysis identified sex specific marker taxa that matched these patterns(Figures 6C,F). *Fusobacteriota* marked the PD_Female group. *Patescibacteria*, including *Candidatus_Saccharimonas* (TM7) taxa, marked the PD_Male group. These results indicate sex dependent oral dysbiosis in PD with distinct taxonomic signatures in the PD_Female and PD_Male groups.

### 3.5 Associations between salivary microbiota and clinical phenotypes

**FIGURE 7:**
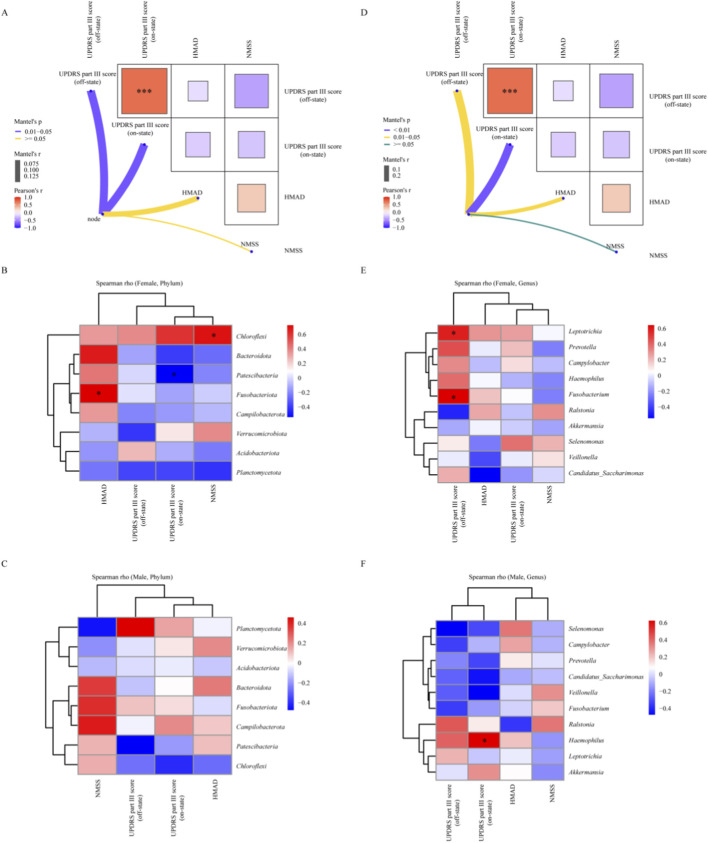
Sex-stratified associations between salivary microbiota and clinical scales in PD. **(A)** Mantel test diagrams for PD_Female group, summarizing correlations between community dissimilarity (Bray–Curtis; salivary microbiome) and distance matrices of clinical measures (UPDRS part III off-state, UPDRS part III on-state, HAMD, NMSS). Arrow width reflects Mantel’s r; color key indicates p levels. Inset matrices show pairwise correlations among clinical scales. **(B,C)** Phylum-level heatmaps for PD_Female group **(B)** and PD_Male group **(C)** showing Spearman’s rho between taxa relative abundance and clinical scales; rows and columns are hierarchically clustered. **(D)** Mantel test diagram for the PD_Male group, with the same statistical framework and visual representation as in **(A)**. **(E,F)** Genus-level heatmaps for D_Female group **(E)** and PD_Male group **(F)**, as in **(B,C)**. Asterisks denote significance thresholds (p < 0.05) where applicable.

Sex-stratified correlation analyses related community dissimilarity to clinical scales. Mantel tests showed the strongest associations with motor severity. The association with UPDRS part III in the on medication state was stronger than in the off medication state. Associations with the HMAD and NMSS were weaker (Figures 7A, 6D). At the phylum level, the PD_Female group showed a positive correlation of *Fusobacteriota* with HAMD scores, a negative correlation of *Patescibacteria* with UPDRS part III in the on medication state, and a positive correlation of *Chloroflexi* with the NMSS (Figure 7B). In the PD_Male group, no phylum level correlations remained significant after false discovery rate correction (Figure 7C). At the genus level, the PD_Female group showed positive correlations of *Leptotrichia* and *Fusobacterium* with UPDRS part III in the off medication state (Figure 7E). The PD_Male group showed a positive correlation of *Ralstonia* with UPDRS part III in the on medication state, and no other genera reached significance after correction (Figure 7F). Taken together, the phylum and genus level results indicate sex dependent oral dysbiosis. The PD_Female group shows an anaerobe rich and inflammophilic niche. The PD_Male group shows a TM7 skewed niche with features of biofilm remodeling.

## 4 Discussion

This study evaluated the salivary microbiome in PD with explicit sex stratification. Overall α-diversity was largely preserved, though richness differed by sex, being higher in the PD_Male group than in the PD_Female group. β-diversity revealed modest but significant group-level separation across feature, phylum, and genus levels, with explained variance of approximately thirteen percent—consistent with a complex, multifactorial phenotype. These microbial community differences paralleled the clinical data, where PD_Male participants exhibited higher motor burden, whereas PD_Female participants showed greater non-motor and affective symptoms. Beyond statistical differences, several biological themes emerge from these findings. The enrichment of *Prevotella*, *Veillonella*, and *Fusobacterium* in PD_Female participants suggests a shift toward anaerobic, acidogenic metabolism that can promote mucosal inflammation and epithelial stress. These genera are known contributors to periodontal dysbiosis and have been associated with elevated proinflammatory mediators in oral and gut ecosystems. In contrast, reduced *Akkermansia*, a genus important for mucin degradation and epithelial barrier maintenance, may indicate compromised mucosal integrity and heightened susceptibility to inflammation.

In PD_Male participants, the expansion of TM7-related taxa, including *Candidatus Saccharimonas*, aligns with the epibiotic lifestyle characteristic of *Saccharimonadaceae*, which can remodel host-associated biofilms and induce epithelial stress responses. These microbial features indicate distinct ecological pressures in male and female oral niches, possibly reflecting sex-specific immune, hormonal, or metabolic influences. The clinical relevance of these ecological patterns is supported by the observation that microbiome–phenotype associations were stronger for motor severity than for non-motor symptoms, suggesting that oral microbial structure may more closely track neurodegeneration-related physiological changes. Similar themes have been reported in gut microbiome studies in PD, where anaerobe-enriched communities and TM7 expansion have been linked to intestinal inflammation and dopaminergic pathway disruption (Rei et al., 2024; Song et al., 2023).

Our findings are consistent with previous evidence that the PD oral microbiome shifts toward an anaerobe-dominated, inflammation-prone profile characterized by enrichment of *Prevotella* and *Veillonella* and depletion of *Neisseria* and *Haemophilus* (Clasen et al., 2025; Murcia-Flores et al., 2025), and that these changes may contribute to mucosal inflammation and plaque dysbiosis (Rajasekaran et al., 2024). The present study extends this body of work by demonstrating that these alterations are not uniform across sexes. PD_Female participants exhibited an anaerobe-oriented community with reduced *Akkermansia*, a genus central to mucin maintenance and epithelial barrier function, whereas PD_Male participants showed more prominent TM7-related signatures, which are known to contribute to mucosal stress and periodontal inflammation (Li et al., 2025).

Together, these results integrate established gut and oral microbial patterns within a sex-stratified framework and provide direct evidence for sex-specific ecological remodeling of the salivary microbiome in PD. Coupling between microbial community structure and clinical phenotype was strongest for motor severity. The correlation with UPDRS part III in the on-medication state exceeded that in the off state, while associations with NMSS were weaker. These findings suggest that oral microbial patterns more closely reflect motor symptom burden. In PD_Female participants, Leptotrichia and *Fusobacterium* correlated positively with UPDRS part III (off state), while in PD_Male participants, Ralstonia correlated positively with UPDRS part III (on state). These data reinforce the notion that sex modulates the link between salivary microbiota and clinical phenotypes (Schaffner et al., 2025), echoing reports connecting anaerobe-rich oral communities and TM7 expansion to inflammatory tone and biofilm remodeling (Peng et al., 2022).

Biological interpretation should remain cautious. In the PD_Female group, enrichment of Prevotella, Veillonella, and *Fusobacterium* may promote acidogenic metabolism and mucosal inflammation, while reduced Akkermansia could reflect compromised mucosal integrity (Song et al., 2023; Mo et al., 2024). In PD_Male participants, the increase in TM7-related taxa and Ralstonia may indicate biofilm remodeling and epithelial stress (Romano et al., 2025). These hypotheses warrant validation using functional and mechanistic assays.

Limitations of this study include modest effect sizes and the cross-sectional design, which does not allow determination of temporal or causal relationships. Important oral health and medication-related variables such as plaque index, salivary flow rate, oral hygiene habits, dietary patterns, and the use of proton pump inhibitors were not systematically documented. Although participants with acute oral infections, recent major dental procedures, or self-reported severe periodontal disease were excluded after a brief oral examination, comprehensive periodontal assessments were not performed. As a result, residual confounding from chronic oral conditions cannot be excluded. Additionally, 16S rRNA sequencing provides relative rather than functional or strain-level information, and the absence of negative controls means that low-level contamination cannot be fully ruled out. The single-center design and moderate sample size may also limit the generalizability of the findings.

Despite these limitations, the study has several strengths. Sex-stratified design allowed exploration of clinical heterogeneity. Parallel assessment of motor and non-motor domains under on/off states enhanced interpretability. Methodologically, we integrated α/β-diversity analysis, IndVal validation, and correlation mapping linking taxa with clinical measures. Standardized saliva collection minimized circadian and behavioral confounding.

Future research should extend beyond descriptive microbiome profiling toward functional validation. Longitudinal, multi-center studies with standardized levodopa exposure windows could assess the temporal stability of sex-specific signatures. Multi-omics approaches—such as shotgun metagenomics, metatranscriptomics, and metabolomics—could identify microbial pathways related to lactate metabolism, mucin degradation, and oxidative stress. Integration of salivary cytokine and secretory IgA profiles would further connect microbial ecology with host immune responses.

Importantly, the expanding field of microbial therapeutics and engineering offers promising translational avenues. Engineered microbes have been designed to modulate neurotransmitter signaling, inflammatory tone, and barrier function, suggesting potential applications in PD-associated dysbiosis (Kamble et al., 2025). Synthetic biology could enable the design of salivary probiotics that restore mucin integrity or dampen local inflammation. Microbial engineering has already demonstrated therapeutic utility in other neuroinflammatory conditions (Hemmerling et al., 2023), and similar strategies may 1 day allow targeted restoration of oral–gut–brain homeostasis in PD.

Future work should thus combine sex-aware microbiome mapping with microbial engineering and precision probiotic strategies to explore causal mechanisms and therapeutic modulation.

## 5 Conclusion

PD is associated with sex-dependent alterations of the salivary microbiome. PD_Female participants exhibit an anaerobe-enriched and inflammophilic microbial profile with reduced *Akkermansia*, whereas PD_Male participants display TM7-dominated biofilm signatures suggestive of epithelial stress. Associations between microbial communities and clinical measures are strongest for motor severity. Beyond microbial profiling, this work supports a model where the salivary microbiome reflects systemic and neurological heterogeneity in PD. Emerging microbial engineering approaches may enable targeted manipulation of oral microbial ecosystems to restore mucosal resilience and modulate neuroinflammatory pathways. Validation in larger, longitudinal, and mechanistically focused cohorts will be critical to translating these findings toward biomarker development and microbiota-based interventions.

## Data Availability

The datasets generated and analyzed during the current study are not publicly available due to patient privacy and ethical restrictions, but are available from the corresponding author on reasonable request.
