# Supplementary material for: Sex-dependent alterations of salivary microbiome in Parkinson’s disease: associations with motor and non-motor clinical phenotypes
Source: Front Mol Biosci. 2025 Dec 8;12:1726620. doi: 10.3389/fmolb.2025.1726620 (PMC12722840; doi:10.3389/fmolb.2025.1726620)
Supplement: Supplementary file 1 [file Supplementaryfile1.docx]

**Table S1.** Alpha-diversity indices of salivary microbiota among Parkinson’s disease and healthy control groups stratified by sex.

|  | NC_Female | NC_Male | PD_Female | PD_Male | p | p(NC_Female_vs_NC_Male) | p(NC_Female_vs_PD_Female) | p(NC_Female_vs_PD_Male) | p(NC_Male_vs_PD_Female) | p(NC_Male_vs_PD_Male) | p(PD_Female_vs_PD_Male) |
| --- | --- | --- | --- | --- | --- | --- | --- | --- | --- | --- | --- |
| Sob | 1260±260.3 | 2753±733.4 | 1583±745.4 | 3209±434.1 | 0.0013 | 0.1994 | >0.9999 | 0.0132 | 0.1023 | >0.9999 | 0.0058 |
| ACE | 1521±235.5 | 2982±692.7 | 1840±722.2 | 5304±787.2 | 0.0004 | 0.1323 | >0.9999 | 0.0033 | 0.1023 | >0.9999 | 0.0025 |
| Shannon | 5.73±0.2 | 5.98±0.27 | 6.02±0.14 | 6.05±0.22 | 0.5009 | - | - | - | - | - | - |
| Simpson | 0.93±0.01 | 0.93±0.02 | 0.95±0.01 | 0.92±0.02 | 0.5259 | - | - | - | - | - | - |

Values are presented as mean ± standard deviation. Global comparisons were performed using Kruskal–Wallis non-parametric tests, and pairwise comparisons were conducted with Dunn’s post hoc test.

**Table S2. Relative abundance of salivary microbiota at the phylum level across Parkinson’s disease and healthy control groups stratified by sex.**

|  | NC_Female | NC_Male | PD_Female | PD_Male | p | p(NC_Female_vs_NC_Male) | p(NC_Female_vs_PD_Female) | p(NC_Female_vs_PD_Male) | p(NC_Male_vs_PD_Female) | p(NC_Male_vs_PD_Male) | p(PD_Female_vs_PD_Male) |
| --- | --- | --- | --- | --- | --- | --- | --- | --- | --- | --- | --- |
| Bacteroidota | 20.87±14.64 | 15.22±  6.98 | 22.46  ±8.42 | 12.37  ±5.61 | 0.047 | 0.558 | 0.159 | 0.170 | 0.050 | 0.440 | 0.006 |
| Patescibacteria | 7.34±8.43 | 15.76  ±18.23 | 3.44  ±2.47 | 24.25  ±15.78 | 0.001 | 0.261 | 0.257 | 0.007 | 0.027 | 0.123 | 0.000 |
| Fusobacteriota | 4.28  ±3.59 | 4.05  ±3.05 | 8.53  ±4.43 | 2.04  ±1.77 | 0.002 | .981 | .012 | .182 | .013 | .199 | .000 |
| Campilobacterota | 0.45  ±0.30 | 0.63  ±0.48 | 1.47  ±1.33 | 0.50  ±0.72 | 0.043 | .758 | .036 | .485 | .080 | .324 | .006 |
| Verrucomicrobiota | 0.95  ±1.60 | 0.15  ±0.13 | 0.03  ±0.06 | 0.33  ±0.50 | 0.003 | .804 | .001 | .431 | .002 | .590 | .011 |
| Acidobacteriota | 0.004  ±0.006 | 0.30  ±0.80 | 0.03  ±0.04 | 0.30  ±0.73 | 0.049 | .046 | .294 | .009 | .351 | .544 | .124 |
| Chloroflexi | 0.005  ±0.008 | 0.18  ±0.25 | 0.02  ±0.06 | 0.13  ±0.17 | 0.012 | .015 | .981 | .021 | .019 | .951 | .025 |
| Planctomycetota | 0.002  ±0.005 | 0.12  ±0.35 | 0.03  ±0.05 | 0.13  ±0.29 | 0.018 | .097 | .116 | .002 | .905 | .160 | .119 |

Data are expressed as mean ± standard deviation. Global comparisons were performed using Kruskal–Wallis non-parametric tests, with pairwise differences evaluated by Dunn’s post hoc test.

**Table S3. Relative abundance of salivary microbiota at the** genus **level across Parkinson’s disease and healthy control groups stratified by sex.**

|  | NC_Female | NC_Male | PD_Female | PD_Male | p | p(NC_Female_vs_NC_Male) | p(NC_Female_vs_PD_Female) | p(NC_Female_vs_PD_Male) | p(NC_Male_vs_PD_Female) | p(NC_Male_vs_PD_Male) | p(PD_Female_vs_PD_Male) |
| --- | --- | --- | --- | --- | --- | --- | --- | --- | --- | --- | --- |
| Prevotella | 7.10  ±6.63 | 6.12±3.12 | 15.94±7.82 | 5.68±3.25 | 0.008 | 0.935 | 0.007 | 0.701 | 0.010 | 0.648 | 0.002 |
| Veillonella | 3.56±3.35 | 6.09±5.60 | 7.10±4.39 | 1.61±0.81 | 0.014 | 0.574 | 0.028 | 0.292 | 0.110 | 0.113 | 0.001 |
| Haemophilus | 7.88±7.23 | 3.60±3.74 | 3.68±4.53 | 0.62±0.56 | 0.019 | 0.475 | 0.579 | 0.003 | 0.875 | 0.028 | 0.018 |
| Candidatus_Saccharimonas | 2.05±3.10 | 2.15±1.56 | 0.62±0.55 | 6.32±4.70 | 0.002 | 0.451 | 0.060 | 0.045 | 0.010 | 0.219 | 0.018 |
| Fusobacterium | 2.35±2.59 | 2.39±2.28 | 4.26±2.43 | 1.19±0.89 | 0.037 | 0.894 | 0.043 | 0.399 | 0.063 | 0.338 | 0.005 |
| Leptotrichia | 1.60±1.69 | 1.59±1.47 | 4.77±3.04 | 0.79±1.12 | 0.000 | 0.737 | 0.004 | 0.149 | 0.012 | 0.075 | 0.000 |
| Ralstonia | 0.41±0.79 | 1.96±4.06 | 0.01±0.01 | 6.47±8.70 | 0.001 | 0.460 | 0.050 | 0.053 | 0.008 | 0.239 | 0.000 |
| Selenomonas | 0.44±0.45 | 0.40±0.23 | 0.98±0.81 | 1.57±1.14 | 0.010 | 0.841 | 0.112 | 0.006 | 0.079 | 0.004 | 0.265 |
| Campylobacter | 0.41±0.31 | 0.54±0.43 | 1.57±1.31 | 0.47±0.76 | 0.018 | 0.801 | 0.018 | 0.487 | 0.038 | 0.353 | 0.003 |
| Akkermansia | 1.04±1.65 | 0.09±0.12 | 0.01±0.03 | 0.22±0.40 | 0.015 | 0.169 | 0.001 | 0.287 | 0.074 | 0.787 | 0.044 |

Data are expressed as mean ± standard deviation. Global comparisons were performed using Kruskal–Wallis non-parametric tests, with pairwise differences evaluated by Dunn’s post hoc test.
